# Relationship Satisfaction Reduces the Risk of Maternal Infectious Diseases in Pregnancy: The Norwegian Mother and Child Cohort Study

**DOI:** 10.1371/journal.pone.0116796

**Published:** 2015-01-21

**Authors:** Roger Ekeberg Henriksen, Torbjørn Torsheim, Frode Thuen

**Affiliations:** 1 Centre for Evidence-Based Practice, Bergen University College, Bergen, Norway; 2 Faculty of Psychology, Department of Psychosocial Science, University of Bergen, Bergen, Norway; 3 Centre for Evidence-Based Practice, Bergen University College, Bergen, Norway; University of Rennes-1, FRANCE

## Abstract

**Objectives:**

The aims of this study were to explore the degree to which relationship satisfaction predicts the risk of infectious diseases during pregnancy and to examine whether relationship satisfaction moderates the association between stressful life events and the risk of infections.

**Methods:**

This was a prospective study based on data from the Norwegian Mother and Child Cohort Study (MoBa) conducted by the Norwegian Institute of Public Health. Pregnant women (n = 67,244) completed questionnaires concerning relationship satisfaction and nine different categories of infectious diseases as well as socioeconomic characteristics and stressful life events. Associations between the predictor variables and the infectious diseases were assessed by logistic regression analyses. A multiple regression analysis was performed to assess a possible interaction of relationship satisfaction with stressful life events on the risk for infectious diseases.

**Results:**

After controlling for marital status, age, education, income, and stressful life events, high levels of relationship satisfaction at week 15 of gestation were found to predict a significantly lower risk for eight categories of infectious diseases at gestational weeks 17–30. No significant interaction effect was found between relationship satisfaction and stressful life events on the risk for infections.

## Introduction

This study examines to what degree partner satisfaction predicts the risk for infectious diseases in pregnancy. Maternal infectious disease during pregnancy has been recognized as an important risk factor for adverse foetal outcomes. Influenza during pregnancy is associated with an increased risk of preterm birth and low birth weight [1]. Urinary tract infection has been shown to increase the risk for preeclampsia [2]. Other possible consequences include the harmful impact on foetal development of the central nervous system. Evidence demonstrates that prenatal exposure to maternal influenza and genital infections are associated with an increased risk for schizophrenia [3,4]. Evidence also suggests that maternal infection and immune dysfunction may be associated with autism [4]. In addition to potentially harmful outcomes for mother and child, pregnant women are at an increased risk of hospitalization related to complications from an infectious disease compared with nonpregnant women [1]. Increased knowledge on factors that may reduce the prevalence of infectious diseases during pregnancy is of great importance for individuals as well as for society.

Relationships with family and friends are important factors in explaining inequalities in individuals’ health outcomes. Evidence clearly shows that individuals who perceive their social relationships as satisfying or supportive have better health and longer life expectancies than those who report less perceived support from others [5–7]. In adulthood, romantic relationships have been recognized to be particularly important in predicting health outcomes, and several studies have demonstrated the health benefits of being married compared to living alone [8–10]. Although couple relationships seem to be generally health promoting, research also shows that the advantage of being married depends on the quality of the relationship [11–16]. This applies to a number of diseases and health conditions, including infectious diseases. For instance, Phillips and colleagues [17] demonstrated a positive relationship between a high degree of marital satisfaction and a higher antibody response to influenza vaccination in a sample of elderly individuals. Given that antibody response to vaccination is a reliable indicator of susceptibility to infectious diseases, the study suggests that individuals who score high on relationship satisfaction experience lower rates of infectious diseases.

Several possible mechanisms have been proposed to explain how the quality of romantic relationships may influence health, and a number of studies support that couple relationship quality influences health outcomes through both main effects and moderating effects [18–20]. An adverse main effect may result from the tendency that individuals in low-quality relationships struggle with negative aspects related to spousal hostility and conflicts [21]. As with several other sources of psychosocial stress, marital distress has been linked to impairment of the immune system [22,23]. For instance, in a laboratory-based study, Kiecolt-Glaser and colleagues [23] found that married individuals who demonstrated hostile behaviour during a monitored discussion showed higher serum levels of pro-inflammatory cytokines, including interleukin-6 and tumour necrosis factor, compared to baseline levels. In addition to the direct effects mentioned above, relationship quality may moderate the adverse effects of other stressful experiences. It is well established that stressful life events predict alterations in the immune system and susceptibility to infectious diseases [24–28]. Stressful experiences activate the hypothalamic–pituitary–adrenal–cortical system (HPA axis), which leads to the release of cortisol that, in turn, affects the immune system [29,30]. When exposed to a stressful event, support from the partner may reduce the stress-induced activation of the HPA axis [31]. A recent study found that partner support downregulated the effects of psychological distress on maternal cortisol secretion during pregnancy [32]. Interestingly, it seems that the level of partner-related moderation of stress-responses is a function of relationship quality. It has repeatedly been demonstrated that the stress-buffering effect of partner support tends to be stronger when the relationship quality is high [31–33]. For example, an experiment conducted by Coan, Schaefer and Davidson [31] demonstrates how holding a partner’s hand during a stressful event influences activation in the neural systems supporting emotional and behavioural threat responses. 16 women were confronted with the threat of a mild electric shock while either holding their partners’ hands, holding a stranger’s hand, or alone. When exposed to the threat of shock without the support of handholding, the threat-related brain activity reached its highest level. When holding a stranger’s hand, the level of threat-related activity decreased as compared with receiving no support of handholding. While holding their partners’ hands, the participants showed the lowest levels of threat-related brain activity. Interestingly, the effects varied as a function of marital quality, with higher marital quality predicting less activation in the neuropsychological pathways associated with physiological stress responses.

In summary, the published studies suggest at least two sets of reasons why couple relationship quality may influence susceptibility to infections. First, individuals in the low-quality relationship group are likely to suffer from relational distress and the potentially harmful physiological stress responses that follow. Second, compared with individuals in high-quality relationships, those in low-quality relationships experience less efficient reduction of stress-induced activation in the HPA axis and therefore greater impairment of the immune system’s ability to fight infections. On the basis of the existing literature, we hypothesize that low levels of relationship satisfaction increases the risk for infectious diseases during pregnancy. We further hypothesize that the rate of stressful life events predicts the risk of infectious diseases and that the level of relationship satisfaction moderates this correlation.

Previous research on the general population has provided valuable information on the association between romantic relationship satisfaction and biomarkers that indicate susceptibility to infectious diseases [34]. Corresponding studies on the pregnant population are limited. Previous studies assessing the potentially health-protecting effects of relationship quality in pregnancy have focused on birth outcomes and maternal mental health [35–38]. Therefore, the purpose of the present study is to extend the available knowledge regarding the effects of relationship satisfaction on manifested infectious diseases in pregnancy.

## Materials and Methods

The Norwegian Mother and Child Cohort Study (MoBa) is a prospective population-based pregnancy cohort study conducted by the Norwegian Institute of Public Health [39]. In the period 1999–2008, all (except two) hospitals in Norway with more than 100 births per year invited pregnant women to participate in the study provided they could read Norwegian. They received a postal invitation three weeks before the ultrasound examination routinely offered to all pregnant women in Norway. Of the invited women, 40.6% consented to participate, and the cohort now includes 114,500 children, 95,200 mothers, and 75,200 fathers. Blood samples were obtained from both parents during pregnancy and from mothers and children (umbilical cord) at birth. Follow-up is conducted by questionnaires at regular intervals and by linkage to national health registries. Several sub-studies are conducting additional collections of data and biological materials. The current study is based on version 7 of the quality-assured data files released for research on 17 October 2012. A written informed consent was obtained from each MoBa participant upon recruitment. The study has obtained a licence from the Norwegian Data Inspectorate and was approved by The Regional Committee for Medical Research Ethics.

### Participants

The present study includes married and cohabiting women participating in the MoBa study for the first time (n = 75,730). Thus, multi-time participating mothers were included with only their first participating pregnancy, and mothers living alone were excluded. Among those, 67,244 participants answered all questions of concern for the present study. This means that 8486 participants were excluded from the multivariate analyses due to missing values at one or more of the research variables. The participants had a mean age of 29.6 years (standard deviation [SD] 4.55); 52.1% were married, 47.9% were cohabiting, and the average duration of the couple relationships was 6.3 years (SD 4.33). The average level of education was higher for the sample than for the general population of women aged between 16–49 years (official statistics from 2008 in brackets): compulsory school 7.0% (28.1%), vocational school 26.7% (35.7%), three-year college 39.2% (29.8%), and university/higher education 21.9% (6.4%). The full sample has been compared with the general population of pregnant women in Norway and is described in more detail elsewhere [40].

### Measures

*Relationship satisfaction* (RS) was measured during week 15 of the current pregnancy, using the full 10-items version of the Relationship Satisfaction Scale. The scale was developed for the MoBa and is based on core items used in previously developed measures of marital satisfaction and relationship quality [41–43]. The scale has a six-step scoring format spanning from 1 ‘totally agree’ to 6 ‘don’t agree at all’. Examples of items are: ‘My partner and I have problems in our relationship’; ‘I am very happy in my relationship’; ‘My partner is generally very understanding’; and ‘I am satisfied with my relationship with my partner’. In the current sample the scale had a Cronbach’s alpha of 0.90. This scale has been used in previous studies [37,44]. In the logistic regression analyses, the scale was reversed and indexed to achieve a maximum score of 1 (low RS) and a minimum score of 0 (high RS).

*Infectious diseases* were measured during week 30 of the current pregnancy. The measure is based on a checklist covering nine different categories of infectious diseases: influenza, pneumonia/bronchitis, diarrhea/gastric flu, common cold, throat infection, sinusitis/ear infection, vaginal thrush, vaginal catarrh and bladder infection. The respondents were asked to mark whether they had (= 1) or had not (= 0) experienced the respective disease between weeks 17 and 30 of the current pregnancy. In order to examine the effect of relationship satisfaction and stressful life events on the total number of self-reported infections, a scale was constructed based on sum scores of the reported diseases.

*Stressful Life Events* (SLE) were measured during week 30 of the current pregnancy and consisted of 11 questions concerning different types of demanding experiences. Participants were asked to report whether they had experienced any of the listed situations during the preceding 12 months. Examples of questions are: ‘Have you had financial problems?’, ‘Have you had problems or conflict with your family, friends, or neighbours?’, ‘Has anyone close to you been seriously ill or injured?’, ‘Have you been involved in a serious accident, fire, or robbery?’ and ‘Have you lost someone close to you?’. The scoring format for each question was no (0) or yes (1). A scale was constructed based on sum scores reflecting the number of reported stressful life events, ranging from 0 to 8.

### Control variables

Socioeconomic factors are, in general, known to predict physical health outcomes and were therefore included as control variables. These variables included age, marital status (married versus cohabiting), maternal income (scored from 1 = no income to 7 ≥ NOK 500,000 ≈ EUR 66,000), and educational level (six categories from public school to >4 years at university/college).

### Statistical analyses

All statistical analyses were conducted using IBM SPSS, version 21. The associations between relationship satisfaction and infectious diseases were first tested by performance of separate bivariate logistic regression analyses for each of the nine different infectious diseases as the dependent variable and relationship satisfaction as the predictor variable. In order to assess the unique contribution of relationship satisfaction in the full model, nine additional multivariable logistic regression analyses were performed with the inclusion of the following independent control variables: age, marital status, education, income, and stressful life events. One logistic regression analysis was also conducted with all nine infections collapsed into one dichotomized variable (no infections versus one or more infections). To assess a possible interaction of relationship satisfaction with stressful life events on the number of self-reported infectious diseases, a hierarchical multiple regression analysis was performed by first entering the main predictor variables based on the continuous relationship satisfaction and stressful life events scores, with marital status, age, education, and income included as control variables. An interaction term based on the continuous relationship satisfaction and stressful life events scores was then added. In order to avoid collinearity between the main effects and the interaction term, both variables were centered on their means before multiplied together.

## Results

Descriptive analyses showed that the majority of the sample reported high levels of relationship satisfaction (M = 5.37, SD = 0.61). At least one infectious disease (M = 1.14, SD = 1.54) was experienced by 53.7% of the participants between week 17–30 of the current pregnancy. Stressful life events was experienced by 56.1% of the participants (1 SLE: 30.2%; 2 SLEs: 16.9%; 3 SLEs: 6.5%; 4 SLEs: 2.0%. Only 0.6% of the participants reported 5 or more SLEs). The mean number of reported stressful life events was 0.95 (SD = 1.07), ranging from 0 to 8. The results from the bivariate and multivariate logistic regression analyses and the prevalence of each disease are displayed in Table 1. The bivariate analyses showed a significant association between relationship satisfaction and eight of the infectious diseases, with odds ratios varying between 1.5 and 4.3 (95% CI). After adjusting for scores on stressful life events the odds ratios of the eight infections varied between 1.4 and 3.4, a decrease by an average of 0.38 (SD = 0.25) as compared with the unadjusted ORs. After adjusting for marital status, age, income, education, and stressful life events, the level of relationship satisfaction was still significantly associated with eight of the disease variables, with odds ratios varying between 1.6 and 3.3 (95% CI). When all nine groups of infections were collapsed into one dichotomized variable, the odds ratio was 2.1 (95% CI 1.86–2.40) in the bivariate analysis, 1.9 (95% CI 1.63–2.10) when adjusting for stressful life events, and 2.0 (95% CI 1.74–2.25) when adjusting for stressful life events, marital status, age, income, and education.

**Table 1:** Associations between relationship satisfaction^a^ and self-reported infectious diseases.

**Infections**	**Prevalence**	**OR unadjusted 95% CI**		OR adjusted * 95% CI	
	% of n = 67,244	Exp (B)	(Lower–Upper)	Exp (B)	(Lower–Upper)
Influenza	5.7	2.5	(1.98–3.25)	2.4	(1.81–3.01)
Pneumonia/bronchitis	1.9	2.5	(1.65–3.82)	2.0	(1.27–3.01)
Common cold	33.5	1.5	(1.36–1.76)	1.6	(1.38–1.80)
Throat infection	3.4	3.4	(2.51–4.61)	2.8	(2.01–3.80)
Sinusitis/ear infection	5.0	2.9	(2.24–3.75)	2.4	(1.85–3.15)
Diarrhea/gastric flu	11.7	2.5	(2.07–2.97)	2.3	(1.93–2.80)
Vaginal thrush	19.8	2.1	(1.77–2.40)	1.8	(1.54–2.10)
Vaginal catarrh	3.9	4.3	(3.26–5.69)	3.3	(2.51–4.46)
Urinary bladder infection	5.1	1.1	(0.81–1.43)	0.9	(0.69–1.23)
**At least one infection**	**53.7**	**2.1**	**(1.86–2.40)**	**2.0**	**(1.74–2.25)**

* Odds ratios were adjusted for age, level of education, income, marital status, and stressful life events

Note. a. Index from 0 (high RS) to 1 (low RS).

A hierarchical multiple regression analysis was conducted that examined the interaction-effect of relationship satisfaction and stressful life events on the total number of self-reported infectious diseases. Model 1 included RS and SLE as the main predictor variables and age, income, education and marital status as control variables. The model explained 1.6% of the variance (adjusted R^2^ = .016, F(6,67237) = 182.494, p<.001). There was a significant positive association between the level of stressful life events and infectious diseases (unstandardized B = .125, standardized β = .087, p<.001) and a significant negative association between relationship satisfaction and infectious diseases (unstandardized B −.174, standardized β = −.069, p<.001). In Model 2 the interaction term RS × SLE was added. No statistically significant interaction effect was found between relationship satisfaction and stressful life events on the risk of infectious diseases (unstandardized B = −.015, standardized β = −.007, p= .066). By adding the interaction term, the R square did not change significantly (R^2^ change < 0.0001, F(1,67236) = 3.382, p = .066), and the explained variance of the model remained 1.6% (R^2^ = .016, F(7,67236) = 156.912, p<.001).

## Discussion

The present study examined the associations between relationship satisfaction and nine different groups of self-reported infectious diseases among married and cohabiting women in mid-pregnancy. The results from the logistic regression analyses showed that relationship satisfaction measured in week 15 of pregnancy is significantly associated with eight out of nine infectious diseases experienced between weeks 17–30 of pregnancy. The results persisted after controlling for scores on marital status, age, income, education, and stressful life events. Among the infections assessed in this study, only urinary bladder infection was not significantly associated with relationship satisfaction. All other groups of infectious diseases (influenza, pneumonia/bronchitis, diarrhea/gastric flu, common cold, throat infection, sinusitis/ear infection, vaginal thrush, and vaginal catarrh) were significantly negatively associated with relationship satisfaction. The data available for this study do not provide an explanation why urinary bladder infection did not follow the same pattern as the other infections. However, it is well documented that high frequency of sexual intercourse is the most important risk factor for urinary tract infections [45]. A possible explanation for the negative finding in the present study might be that woman reporting poor relationship satisfaction are less sexually active and therefore not exposed to the same risk for bladder infections than their more satisfied counterparts.

Overall, the odds of experiencing an infection for pregnant women with low relationship satisfaction were twice as high compared with pregnant woman reporting high relationship satisfaction. The results support our hypothesis that the level of relationship satisfaction predicts the risk for infectious diseases in pregnancy. These findings are noteworthy because infectious diseases have a potential for harming both the mother and the developing foetus when occurring during pregnancy [1–4]. Because the present study did not measure couple behaviour or neuropsychological responses related to relationship satisfaction, we cannot conclude how relationship quality influences the risk of infections. However, based on previous research in the general population the present results may be partly explained by the direct immune-impairing effects of hostility and conflicts that more frequently occur in dysfunctional relationships [21,23]. It has also been suggested that partner support may be beneficial because it decreases biological sensitivity to psychological distress [32]. Indeed, emotion-regulation has been linked to activation of the HPA axis and researchers have suggested that emotion-regulation may be a key factor in links between marital quality and health [19,32,46,47]. Anyhow, the direct effect of relationship satisfaction on the level of self-reported infections found in the present study do harmonize with previous research showing that marital quality is linked to biomarkers of stress known to impair immune function [48,49]. Also in line with previous studies [24–28], the present results show that stressful life events significantly predicted the risk of infectious diseases.

The hypothesis that relationship quality moderates the adverse effects of stressful life events was not supported by the results. Contrary to our expectations, the results showed a very weak interaction coefficient at a non-significant level between relationship satisfaction and stressful life events on the number of reported infectious diseases. Our hypothesis of interaction was based on previous research indicating that partner support has a moderating effect on biomarkers associated with stress and immune function, such as cortisol and pro-inflammatory cytokines [15,29,32,50]. For example, it was recently demonstrated that effective partner support during pregnancy has the potential to reduce maternal neuroendocrine responses to negative emotional experiences [32]. It should be noted that the present findings are not contradictive to these studies. As we did measure clinical endpoints and not changes in mediating biomarkers, we cannot report on whether relationship quality did moderate stress-related responses or not. We can only conclude that there seem to be no prominent interaction-effect between marital satisfaction and stressful life events on the level of self-reported infections. This is currently a noteworthy finding because the present study is among the first to investigate such a potential interaction-effect on clinical endpoints [34]. This is also the first population-based study to assess the direct association between relationship satisfaction and manifested infectious disease during pregnancy [34]. More comprehensive studies are needed to illuminate how and to what degree the quality of close relationships may moderate the clinical health consequences of chronic stress, as well as documenting central causal pathways between relationship quality and infections [19,51]. One idea would be to utilize blood samples from the current cohort study, and examine immune-related parameters such as cytokines, lymphocytes functions, or telomere length to look for correlations with the present data. This would allow us first to examine whether relationship satisfaction and stressful life events has direct effects on physiological processes and then to examine to what degree individuals who exhibit physiological changes are more likely to develop infectious diseases.

A strength of the present study is its large number of participants; this allows for assessing relative small effects within a narrow confidence interval. Nevertheless, these data must be interpreted with caution. One important limitation is the study’s reliance on self-report questionnaires as the data may be subject to reporting bias. In this respect, the validity and reliability of the outcome measure might be of particular concern. For instance, we cannot know whether the participants based their self-reported diseases on medical diagnoses or self-assessments of experienced symptoms. Moreover, because the respondents were not asked how many times each disease occurred during the 14 weeks period assessed, the current study did not distinguish between single and multiple occurring infections. Nor was it distinguished between mild infections and long-lasting severe infections. It should also be mentioned that the scores of the main study variables were not normally distributed. In small samples this may lead to problems with the interpretation of the statistical tests used in this study. In large samples, however, non-normality of the residuals does not violate the interpretation of significance tests or confidence intervals [52]. Although it may be a concern that only 40% of the invited women consented to participate, a potential impact of selection bias on exposure and outcome variables was evaluated in a previous study [40]. Testing eight different exposure–outcome associations, the authors found no statistically significant differences in association measures between participants in the MoBa and the total population. This indicates that the generalizability of this study is not violated by selection bias.

To conclude, the current study contributes toward a better understanding of the association between relationship quality and manifested infectious disease. The main findings indicate that women with the lowest relationship satisfaction experience about twice as many infectious diseases than women with the highest relationship satisfaction. Although the current findings seem to be clinically interesting it remains to examine how, for whom, and to what degree relationship management and improvement of relationship quality may lead to less infectious diseases [34]. Because very few studies describe associations between relationship quality and clinical endpoints [34], future studies should replicate our findings with the use of subjective self-reports as well as medical diagnoses as outcome variables. To document the causal pathways between relationship quality and infections, a longitudinal study including both biomarkers and clinical endpoints is recommended. Another important next step will be to examine the associations between maternal relationship satisfaction and postpartum outcomes.
